# Beat their swords into ploughshares

**DOI:** 10.1111/1751-7915.12251

**Published:** 2015-01-28

**Authors:** John van der Oost

**Affiliations:** Laboratory of Microbiology, Wageningen UniversityWageningen, Netherlands

The struggle for life has been the main driving force in natural evolution. Billions of years of competition and warfare between viruses, prokaryotes and eukaryotes (in any combination) have shaped the present world. Among these violent encounters, the attack of bacteria by viruses is probably the most frequently occurring host–parasite interaction on our planet. The offensive bacteriophages are not only highly abundant; they also have an extremely efficient proliferation mechanism with very high mutation rates. This imposes a major pressure on the bacteria to continuously develop and adjust appropriate defence strategies, and implies that the co-evolution of bacterial defence systems and viral offence systems proceeds with a very high pace (Stern and Sorek 2011).

To counteract invasions by viruses, different bacteria possess a different set of innate defence systems, including restriction/modification (R/M) systems, toxin/anti-toxin (T/A) systems and abortive infection systems (Makarova *et al*., 2011). Recently, a prokaryotic variant of Argonaute, the slicer nuclease of the eukaryotic ribonucleic acid interference (RNAi) system, has been added to that list of general bacterial immune strategies (Swarts *et al*., 2014). A breakthrough has been the discovery of adaptive immunity in bacteria and archaea. The clustered regularly interspaced palindromic repeats, and associated proteins (CRISPR-Cas) system acquires short deoxyribonucleic acid (DNA) sequences from mobile genetic elements (MGE), and stores them in CRISPR arrays in the host genome. Upon an infection by a previously encountered MGE, the CRISPR memory is expressed as small CRISPR RNAs that guide effector proteins to complementary invading nucleic acids, eventually neutralizing the invasion (Barrangou and Marraffini, 2014; Van der Oost *et al*., 2013).

The arsenal of warfare systems from bacteria and phages has boosted the development of molecular biology. Restriction/modification systems were first discovered in the 1960s. The subsequent adaptation of viral offence and bacterial defence is a beautiful example of a biological arms race (Labrie *et al*., 2010). Apart from fundamental insights, restriction enzymes have allowed for an essential step in the development of molecular genetics research in general, and for biotechnological applications in particular (Roberts, 2005).

Two examples of repurposing bacterial virulence systems include the invasion machinery of plant pathogens. The pTi plasmid from *Agrobacterium tumefaciens* is used as general DNA delivery system in plants and fungi, whereas a *Xanthomonas*-derived transcription activator-like (TAL) effector have been fused to a restriction enzyme resulting in sequence specific TAL effector nucleases (TALEN).

Recently, in depth molecular analyses have triggered the revolutionary development of CRISPR-Cas-derived engineering tools. Cas9 is a relatively simple variant CRISPR-Cas effector complex that can be functionally expressed in a wide range of prokaryotic and eukaryotic cells. Importantly, the RNA guide of Cas9 can easily be manipulated to specifically target any sequence of interest. Although adjusting the specificity for a certain target gene is also possible with the TALEN system, a drawback of this system is that this requires laborious protein engineering. In case of Cas9, only a short oligonucleotide has to be generated and cloned, saving time and money. Applications of the Cas9 system include general genetic engineering (disruption, repair and integration of genes), control of gene expression (stimulation and silencing) and gene labelling (imaging). Coexpression of Cas9 with different guides allows for multiplexing, for instance generating multiple knockouts simultaneously (reviewed by: Charpentier and Doudna, 2014; Van der Oost, 2013; Barrangou and Marraffini 2014; Hsu *et al*., 2014).

**Fig 1:**
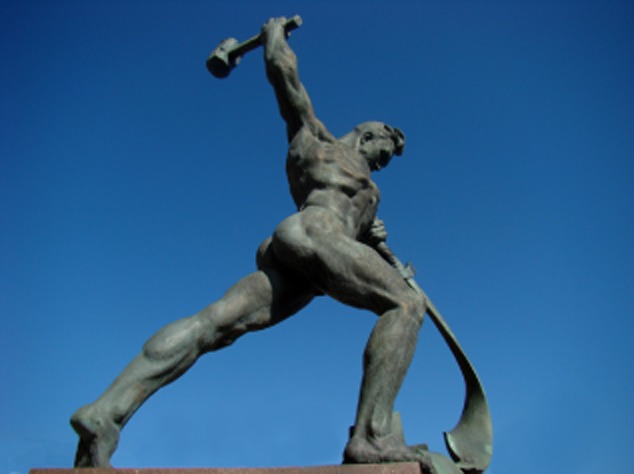
Let us beat Swords into Ploughshares. A sculpture from Evgeniy Vuchetich, inspired by the famous phrase from the Book of Isaiah (‘they shall beat their swords into plowshares, and their spears into pruning hooks’) in front of the United Nations Building in New York. Adjusted from a photo by Gilmar Mattos (https://www.flickr.com/photos/gijlmar/5720499345/in/photostream/).

The currently known virulence and immune systems appear to be just a tip of the iceberg. A recent comparative analysis of prokaryotic genomes has revealed the existence of so-called defence islands: chromosomal gene clusters that encode both integrated MGEs (prophages) and defence systems (R/M, T/A, CRISPR-Cas, Argonaute) (Makarova *et al*., 2011). In addition, these genomic loci contain numerous hypothetical genes, many of which have been predicted to encode novel defence systems. Hence, defence islands provide abundant material for discovering bacterial and archaeal antivirus defence systems. Partly based on the latter predictions, a novel family of viral exclusion systems has recently been characterized (BREX) (Goldfarb *et al*., 2014). Other hypothetical defence systems concern variant Argonautes (reviewed by Swarts 2014) and variant CRISPR-Cas systems (Makarova *et al*., 2013). In conclusion, it seems like a safe bet to predict that there are still many offence and defence systems hidden in microbial genomes. The challenge we are facing is not only to discover and characterize these secret weapons but also explore possibilities to transform them into innovative applications (Fig. 1): beat their swords into ploughshares!
